# A multi-disciplinary, multimodal approach for the management of vascular anomalies

**DOI:** 10.12669/pjms.36.ICON-Suppl.1710

**Published:** 2020-01

**Authors:** Aqsa Mazhar, Shazia Moosa, Alizeh Abbas, Yousuf Mallick, Lubna Samad

**Affiliations:** 1 Aqsa Mazhar, FCPS. Center for Essential Surgical and Acute Care, Global Health Directorate, Indus Health Network, Karachi, Pakistan; 2 Shazia Moosa, MBBS. Center for Essential Surgical and Acute Care, Global Health Directorate, Indus Health Network, Karachi, Pakistan; 3 Alizeh Abbas, MBBS. Center for Essential Surgical and Acute Care, Global Health Directorate, Indus Health Network, Karachi, Pakistan; 4 Yousuf Mallick, FCPS. Department of Dermatology, The Indus Hospital, Karachi, Pakistan; 5 Lubna Samad, MRCS, FCPS. Department of Pediatric Surgery, Center for Essential Surgical and Acute Care, Global Health Directorate, Indus Health Network, Karachi, Pakistan, The Indus Hospital, Karachi, Pakistan

**Keywords:** Vascular malformations, Vascular anomalies, Hemangiomas, Propranolol, PDL (Pulsed dye laser), VAC (Vascular Anomalies Center)

## Abstract

**Objective:**

Vascular anomalies are a diverse group of lesions, ranging from simple to complex, disfiguring anomalies. Our objective was to diagnose and provide comprehensive treatment to patients presenting with vascular anomalies, using a multi-disciplinary approach involving dermatologists, plastic surgeons, radiologists and pediatric surgeons.

**Methods:**

Patients presenting with vascular anomalies to The Indus Hospital, Karachi, from January 2017 to March 2019 were enrolled, using a pre-defined questionnaire. Assessment, diagnostic work up, management and clinical and photographic follow up was maintained to monitor outcomes.

**Results:**

One hundred eighty seven patients with a mean age of 4.6 years, (females 62%) were enrolled. Diagnoses included vascular tumors (n=89, 47.6%), lymphatic malformations (n=38, 20.3%), capillary malformations (n=19, 10%), venous malformations (n=16, 8.5%), arterio-venous malformations (n=14, 7.5%) and mixed anomalies (n=11, 5.9%). Treatment modalities, in isolation or combination, included oral propranolol, topical timolol, pulsed dye laser and intra-lesional sclerotherapy. Mean follow up was in 7.1 months, with 27 patients achieving treatment completion. 26 children were lost to follow-up.

**Conclusions:**

Vascular anomalies have mostly been managed successfully at VAC using single or multimodal treatment. Increasingly complex anomalies can be handled using a multi-disciplinary approach. Establishment of VAC has facilitated many patients who were earlier considered as diagnostic and therapeutic challenges.

## INTRODUCTION

Vascular anomalies are a heterogeneous group of lesions resulting from abnormal vascular development, most commonly involving the cranio-facial region. Often grouped together, vascular tumours and vascular malformations must be categorized separately as described by Mulliken and Glowacki, with the former growing by cellular hyperplasia and the latter representing localized defects in vascular morphogenesis.^1^-^3^

Hemangiomas are benign vascular tumors with ‘Infantile hemangiomas (IH)’ as the predominant variety; IHs are the commonest tumours of infancy, affecting approximately 5-10% of the infantile population.^4^,^5^ Identifiable risk factors include female sex, prematurity, low birth weight, fair skin, positive family history, older maternal age, placental abnormalities and multiple gestations.^6^,^7^ IHs have three discrete phases: proliferative, involuting and involuted; they are not apparent at birth, but almost always present by the first week of life, achieving complete involution by seven to nine years of age.^5^ IHs are sub-classified as focal, multifocal, segmental and indeterminate depending on their morphology, extent or distribution and as superficial, deep and mixed depending on their location in the skin. Segmental IHs may be associated with other anomalies and syndromes.^6^ ‘Congenital hemangiomas’, the uncommon type, could be rapidly involuting (RICH) or non-involuting (NICH).^8^ RICH are present at birth and amenable to regress spontaneously in a few months while NICH do not involute and may need intervention during childhood.

Vascular malformations, on the other hand, infiltrate normal tissue leading to soft tissue destruction; they are non-familial, sporadic and congenital, although they may not be clinically apparent until adolescence or adulthood. Vascular malformations are subcategorized by their predominant channel type (capillary, venous, arterial, lymphatic, or a combination). Based on differences in biological and radiological characteristics, they are further classified into slow-flow and fast-flow lesions^2^ (Table-I). Venous malformations (VM) are the most common type of vascular malformations with an incidence of two per 10,000 live births, followed by lymphatic (LM), capillary (CM) and arteriovenous malformations (AVM).^9^-^11^

**Table I:** Classification of vascular anomalies.

Vascular Tumors	Vascular Malformations
1. Infantile hemangioma	1. Slow-flow
2. Congenital hemangioma	- Capillary malformations (CM)
- Rapidly involuting (RICH)	- Venous malformations (VM)
- Non-involuting (NICH)	- Lymphatic malformations (LM)
3. Tufted angioma	- Mixed malformations
4. Kaposiform hemangioendothelioma	2. Fast-flow - Arteriovenous malformation (AVM)

Patients with vascular anomalies are “medical nomads” as they pose diagnostic and therapeutic challenges owing to diverse presentations, growth patterns, association with anomalies, variable response to treatment and possibility of multi-organ involvement. These complexities warrant a multimodal and multidisciplinary approach with collective efforts of dermatologists, interventional radiologists, pediatricians, plastic surgeons and other specialists to aid in the diagnosis and management of these patients. Comprehensive vascular anomalies care has been lacking in Pakistan, especially in Sindh and Baluchistan. Furthermore, the need for expensive scans, prolonged drug therapy, laser equipment and expertise makes treatment unaffordable to many.

The Indus Hospital (TIH) Karachi, initiated a free of cost, facility-based treatment program with establishment of the Vascular Anomalies Center (VAC) in January 2017. This program is jointly conducted by the Pediatric Surgery and Dermatology departments, with involvement of other specialties as required. The objective is to provide comprehensive treatment to patients presenting with vascular anomalies, through a multi-disciplinary approach.

## METHODS

A retrospective review of all patients enrolled at the VAC between January 2017 and March 2019 was conducted. A pre-defined questionnaire was used to collect demographic and clinical data including age, sex, ethnicity, skin type, presenting complaints, family history and physical findings on initial presentation. Relevant laboratory and radiological findings were also documented following which a final diagnosis was made and management was planned accordingly. Treatment for IH was initiated in case of rapid growth, large size (more than five cm), head and neck region involvement, multiple hemangiomas or in those who failed to enter involution phase. Before initiating oral propranolol in hemangioma patients, Electrocardiography (ECG) was done, followed by echocardiography if any abnormalities were noted in the ECG.

Oral propranolol, topical timolol, pulsed dye laser (PDL) therapy and intra-lesional injection with bleomycin or steroids were used to treat hemangiomas. Intra-lesional injection with bleomycin or steroids, PDL and surgical excision were the options for treating vascular malformations, depending on their type. Patients were evaluated periodically using clinical parameters such as color and size of lesion and comparison of photographic record at each visit. Microsoft Excel 2016 was used to enter data. All patient records were assessed in detail and descriptive analysis was conducted with mean, standard deviation, frequencies and percentages. Enrollment data was kept confidential, accessible only to the primary research team. Ethical approval was obtained from the IRB (IRD_IRB_2018_11_001 Dated on November 8, 2018), then modification was approved from IRB (IRD_IRB_2018_11_001 Dated on May 8, 2019).

### Inclusion Criteria

All patients diagnosed with vascular anomalies.

### Exclusion Criteria

Patients with lesions other than vascular anomalies.

## RESULTS

Mean age of patients was 4.6 years (SD: 6.9 years, median: 1.6 years, range: one month to 39 years), with female preponderance (62%). Prevailing skin type was brown (n= 160, 85.5%); rest were fair skin type (n=27, 14.4%). Primary site of involvement was the head and neck region (n=116, 62%), followed by extremities (n=44, 23.5%) and trunk (n=27, 14.4%). Two patients had associated skeletal deformities and five, with lymphatic malformation involving the tongue, had eating difficulties. A family history of vascular lesions was found in four (2%) patients. Mean follow-up duration was 7.1 months (SD: 6.21 months, range: one - 30 months), with 27 patients achieving treatment completion by March 2019. During this time 26 (14%) patients were lost to follow-up.

**Fig.1:**
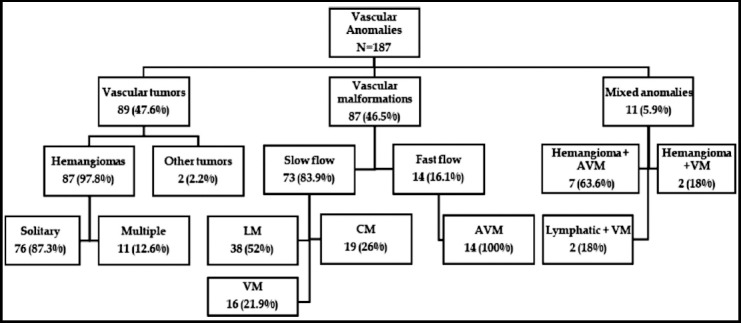
Classification and incidence of vascular anomalies in this study.

The different types of vascular anomalies in our patients are detailed in Fig.1 showing nearly equal incidence of vascular tumors and malformations. Mixed anomalies were seen in 5.9%, with a combination of tumor and malformation or multiple types of vascular malformations presenting in one patient. Treatment modalities and response in patients with hemangiomas and vascular malformations, either in isolation or combination, are shown in Table-II and III respectively.

**Table II:** Treatment modalities and response in hemangiomas.

Single Treatment (n=61)	Prop	IST (B)		I/L (C)	PDL	Timolol		Surgical Excision	Awaiting treatment	Overall response

	41 (67.2%)	4 (6.5%)		3 (4.9%)	1 (1.6%)	11 (18%)		1 (1.6%)	4	Fading of lesion in 90% (n=75) and 50–60% reduction in size in 65% (n=54 ) of patients

Combined Treatment (n=22)	Prop + PDL	Prop + IST (B + C)	Timolol + PDL	IST (B) + Timolol	Prop + Timolol + IST (B)	Prop + Timolol	Prop + PDL + IST	Prop + Timolol + PDL		
	7 (31.8%)	3 (13.6%)	3 (13.6%)	1 (4.5%)	1 (4.5%)	4 (18.1)	2 (9%)	1 (4.5%)		

Prop = Propranolol, IST (B) = Injection sclerotherapy (Bleomycin), I/L (C) = Intra-lesional (corticosteroid), PDL = Pulsed dye laser *Dosage: * Propranolol: 2.5 mg /kg/day in 3 divided doses; timolol: 0.5% gel twice a day;

Inj Bleomycin: 1mg /kg diluted with normal saline (1:1), Inj corticosteroid: 1mg /kg/dose.

**Table III:** Treatment modalities and response in vascular malformations.

Type	IST (Bleomycin)	I/L (STD)	PDL	Surgical Excision	PDL + IST (B)	Awaiting treatment	Response
LM (n=38)	31 (81.5%)	-	-	1 (2.6%)	-	6 (15.7%)	28 (90%) ↓size
CM* (n=19)	-	-	15 (78.9%)	-	-	2 (10.5%)	11 (73%) ↓color
VM (n=16)	9 (56.2%)	3 (18.7%)	-	-	1 (6.25%)	3 (18.7%)	9 (69%) ↓size
AVM (n=14)	-	-	-	-	-	14 (100%)	-

* 2 patients needed reassurance only, (STD) = Sodium tetradecyl sulfate 3% (Dosage: 0.5 - 2 ml).

## DISCUSSION

Vascular anomalies were first classified by Virchow in 1876.^10^ Although the classification stood the test of time for nearly a century, it did not allow standardization of treatment of vascular anomalies because of lack of understanding of the natural behavior of lesions and inaccurate terminologies in use. Mulliken and Glowacki proposed a biological classification in 1982 which was based on endothelial characteristics, physical findings, natural history and histology of lesions.^2^ This classification provided greater insight into the nature of these lesions, enabling the development of better diagnostic and treatment modalities; thus it is used routinely in our practice. (Table-I and Fig.1).

Zheng *et al*. stated that vascular anomalies are most commonly found in the head and neck region.^12^ Very *et al*. also reported primarily head and neck region involvement in 84.8% and 66% of patients with hemangiomas and vascular malformations respectively.^13^ These results are consistent with our findings where 62% of lesions were seen in this region. However, Mathes *et al*. reported that the cervico-facial region was involved in only 31% of all vascular anomalies.^14^ In our study, there is a female preponderance of 62% cases of vascular anomalies, comparable to Western population data.^15^ Family history has been seen to play a role in development of vascular anomalies, but currently there is limited data available in literature establishing this link, except for some cases of childhood hemangiomas, cutaneous VM, CM, AVM^16^-^18^; the four patients in our study with a positive family history of vascular lesions were diagnosed with IH.

Hemangioma is the most common infantile tumour in the Western population but it is not as common in Asian countries.^19^,^20^ Almost half the patients in our series were diagnosed to have hemangiomas. Burd *et al*. reported that infantile hemangiomas manifest during the neonatal period in 80% of children^21^, while Mathes *et al*. reported that 33.3% of vascular tumours and 56.5% of vascular malformations occurred at birth.^14^ Our study demonstrates that 80% of vascular tumours were diagnosed in the first month of life and 58% of these had a precursor lesion at birth; on the other hand, 62% of vascular malformations were diagnosed in the first month of life, with 44% noticed at birth. According to literature^9^-^11^, venous malformations are the commonest type of vascular malformations, however we saw lymphatic malformations most frequently. Another study from Pakistan reported the incidence of fast and slow-flow vascular malformations similar to our study.^22^

Monitoring the dose and side effects of oral propranolol was challenging, especially where caregivers were not able to comprehend written and verbal instructions fully. Detailed and repeated counselling of caregivers, in addition to a helpline to address concerns and queries, provided support during the course of treatment. Of the patients that were started on oral propranolol, response was obtained in all patients but dose had to be titrated very gradually in 10 patients to reach the maintenance dose, due to recurrent episodes of diarrhea or hypoglycemia. Two patients that were unable to tolerate oral propranolol due to recurrent chest congestion and upper respiratory tract infections were switched to topical timolol. Overall, fading of lesion in 90% and 50–60% reduction in size in 65% of hemangioma patients was seen as a result of various modes of treatment employed, predominant mode being oral propranolol. Stringari et al.^23^ reported significant improvement in 95% patients on oral propranolol.

Shiels et al.^24^ showed a 100% response in their patients with LM using injection sclerotherapy with bleomycin. In our study, 90% of patients demonstrated a reduction in size by at least 80% after two to three sessions of injection sclerotherapy. Response to PDL treatment was found to be variable for capillary malformations, with 25 to 75% improvement seen in a majority of our patients; similar results have been reported from the region.^25^ A study from Pakistan reported percutaneous sclerotherapy with bleomycin or sodium tetradecyl sulphate (STD) to be safe and effective for the treatment of venous malformations^11^; a similar management approach demonstrated at least 60% reduction in size after two to three sessions of injection sclerotherapy in majority of our patients.

The need for regular visits and a long-term treatment plan, especially for hemangiomas, may have been the reason for some patients being lost to follow-up; geographical proximity is also an important factor in determining the ability to seek treatment from our facility as some patients have to travel very long distances to avail treatment at the VAC.

## CONCLUSION

Vascular anomalies are a diverse group of abnormalities in blood vessel morphogenesis that usually occur perinatally. In our cohort, the most common vascular anomalies are hemangiomas followed by lymphatic, capillary, venous and AV malformations. Correct diagnosis is critical for initiating appropriate treatment. Successful management can be provided with regular follow-up visits and a multidisciplinary, multimodal approach.

### Authors’ Contribution

**AM:** Conceived, designed, did data collection, did analysis and interpretation of data and manuscript writing.

**SM:** Conceived, designed, did data collection and manuscript editing.

**AA:** Editing of manuscript.

**YM:** Data collection.

**LS:** Takes the responsibility and is accountable for all aspects of the work in ensuring that questions related to the accuracy or integrity of any part of the work are appropriately investigated and resolved.
